# Unilateral transforaminal lumbar interbody fusion through a modified hemilateral spinous process-splitting approach

**DOI:** 10.3389/fneur.2023.1274384

**Published:** 2023-12-21

**Authors:** Guanyi Liu, Xiaodi Zou, Yanzhao Dong, Ahmad Alhaskawi, Lihua Hu, Lu Mao, Jun Qian, Jichong Ying, Sahar Ahmed Abdalbary, Olga Alenikova, Yizhong Ma, Hui Lu

**Affiliations:** ^1^Ningbo No. 6 Hospital, Ningbo, China; ^2^Second Affiliated Hospital, Zhejiang Chinese Medical University, Hangzhou, China; ^3^First Affiliated Hospital, School of Medicine, Zhejiang University, Hangzhou, China; ^4^Zhongda Hospital, Southeast University, Nanjing, China; ^5^First Affiliated Hospital of Anhui Medical University, Hefei, China; ^6^Department of Orthopedic Physical Therapy, Nahda University, Beni Suef, Egypt; ^7^Republican Research and Clinical Center of Neurology and Neurosurgery, Minsk, Belarus; ^8^Alibaba-Zhejiang University Joint Research Center of Future Digital Healthcare, Zhejiang University, Hangzhou, China

**Keywords:** spinous process, multifidus muscle, surgical approach, lumbar spine, internal fixation

## Abstract

**Objective:**

To describe unilateral transforaminal lumbar interbody fusion (TLIF) via a modified hemilateral spinous process-splitting (MHSPS) approach and determine its effectiveness.

**Methods:**

Sixty-five consecutive patients with the lumbar degenerative disease who underwent MHSPS TLIF between August 2020 and July 2021 were retrospectively analyzed. Japanese Orthopedic Association (JOA) score and visual analog scale (VAS) scores for back and leg pain were evaluated before surgery and at the last follow-up. Postoperative paraspinal muscle atrophy was evaluated on axial T2-weighted magnetic resonance imaging.

**Results:**

Mean JOA score increased from 13.6 ± 3.21 before surgery to 24.72 ± 3.34 at last follow-up (*p* < 0.001). The mean recovery rate was 68.2% ± 5.68%. Clinical outcome was excellent in 22, good in 35, and fair in 8 patients. The VAS score for low back pain was significantly lower at the last follow-up than before surgery (1.18 ± 0.99 vs. 3.09 ± 1.35; *p* < 0.001). The VAS score for leg pain was also significantly lower at the last follow-up than before surgery (1.13 ± 0.91 vs. 6.61 ± 1.23; *p* < 0.001). The mean paraspinal muscle atrophy rate did not significantly differ between the symptomatic side (6% ± 3.8%) and asymptomatic side (4.8% ± 3.3%) at last follow -up (*p* = 0.071).

**Conclusion:**

MHSPS TLIF is an effective minimally invasive surgical treatment for selected types of degenerative lumbar disease. This technique can achieve effective spinal decompression and interbody fusion. Its advantages include direct and adequate visualization, vast surgical working space, short operation time, and minimal muscle injury.

## Introduction

Transforaminal lumbar interbody fusion (TLIF), first described by Harms et al. (1) in 1982, has become widely accepted as a standard surgical treatment for degenerative lumbar disk disease (2). Advantages of TLIF over posterior lumbar interbody fusion include minimal dural retraction, high surface area and sufficient blood supply for bony fusion, ability to maintain or restore intervertebral body height, and low risk of postoperative radiculitis (3). Although the conventional midline open approach provides good visualization and vast working space, it requires bilateral detachment and retraction of the paraspinal muscles, which may cause low back pain and atrophy of the muscles (4, 5). To mitigate these potential issues, surgeons have developed minimally invasive (MIS) techniques for TLIF. MIS procedures aim to minimize postoperative pain and preserve muscle integrity, and MIS TLIF using a tubular retractor (2) and TLIF via the Wiltse paraspinal approach (6) have been developed as alternatives. Other MIS-TLIF procedures have also been devised for specific and limited indications. Most MIS approaches require longer operation time and sacrifice surgical visualization and working space to minimize paraspinal muscle damage (6, 7). As a result, they are associated with higher risks of incomplete neural decompression and pseudarthrosis than the conventional open midline approach (7).

Watanabe et al. (8) introduced a spinous process-splitting approach for lumbar laminectomy in which the lamina is exposed by longitudinally splitting the spinous process into halves, while the soft tissue attachments to the spinous process are left intact. This procedure offers more expansive surgical working space and optimizes visualization while causing less muscular damage than the conventional approach (8). Because most lumbar degenerative disease patients suffer from unilateral lower extremity pain, bilateral splitting of the spinous process is unnecessary. Chatani et al. (9) described unilateral partial laminectomy using a hemilateral spinous process-splitting (HSPS) approach for lumbar spinal stenosis and reported satisfactory results. With this approach, the spinous process is split in the midline without stripping the attached muscles; then, the lateral half of the spinous process is resected at the base to expose only the ipsilateral lamina (9). This study aimed to describe unilateral TLIF via a modified HSPS (MHSPS) approach and report our experience with it in patients with lumbar degenerative disease and radiculopathy.

## Methods

### Patients

Clinical data from consecutive patients with unilateral symptoms who underwent MHSPS TLIF between August 2020 and July 2021 were retrospectively reviewed. A single experienced orthopedic surgeon performed all operations. The study protocol was approved by the Ethical Review Board of Ningbo No. 6 Hospital.

Patients who met the following criteria were eligible for inclusion: (1) diagnosis of lumbar degenerative lumbar disk herniation, stenosis, or spondylolisthesis in conjunction with unilateral lumbar radiculopathy; and (2) failure of at least 3 months of conservative treatment. We excluded patients with bilateral lower extremity symptoms, scoliosis, a history of previous lumbar spine surgery, and patients less than 18 years old or less than 12 months of follow-up. Those with incomplete data were also excluded.

### Surgical technique

After induction of general anesthesia, patients were positioned prone on a radiolucent frame. Anteroposterior fluoroscopy was performed to mark the surgical level(s). A midline incision was made, and the spinous processes and interspinous ligaments cranial and caudal to the surgical level(s) were exposed. The subcutaneous tissue on the asymptomatic side was separated from the surface of the lumbodorsal fascia. The Wiltse interval between the medial multifidus and lateral longissimus muscles was identified and bluntly separated. Pedicle screws (Guanlong Co., Ltd., Jinan, Shandong, China) were then inserted.

**Figure 1:**
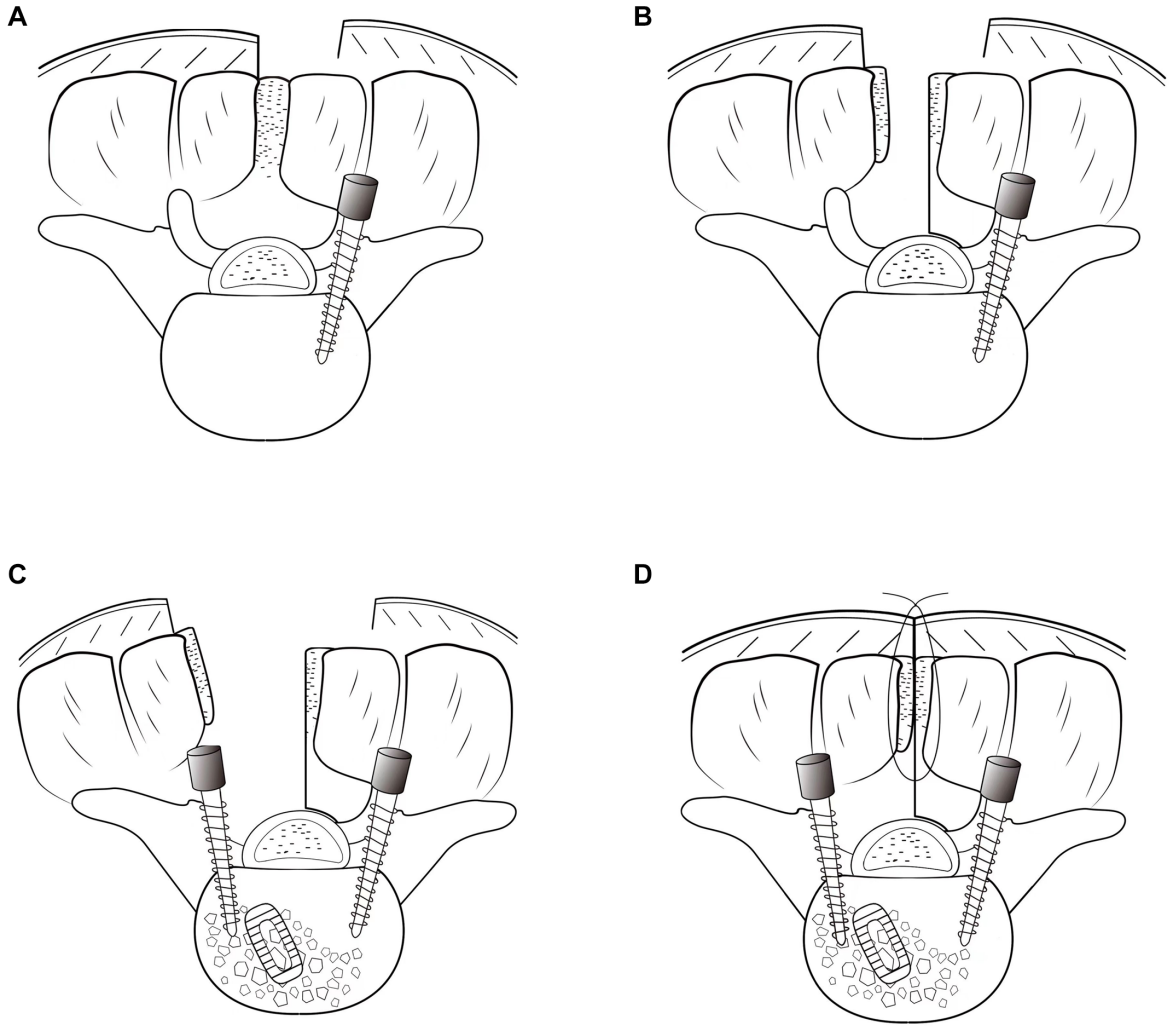
Illustration of the hemilateral spinous process-splitting transforaminal lumbar interbody fusion procedure. Pedicle screws on the asymptomatic side were placed using a Wiltse approach **(A)**. The spinous process was split in the midline with the attached muscles left intact and a hemilateral half of the spinous process for the decompression side was broken **(B)**. After unilateral decompression and interbody fusion were achieved, pedicle screws on the symptomatic side were inserted through the surgical field **(C)**. The spinous process was reattached **(D)**.

**Figure 2:**
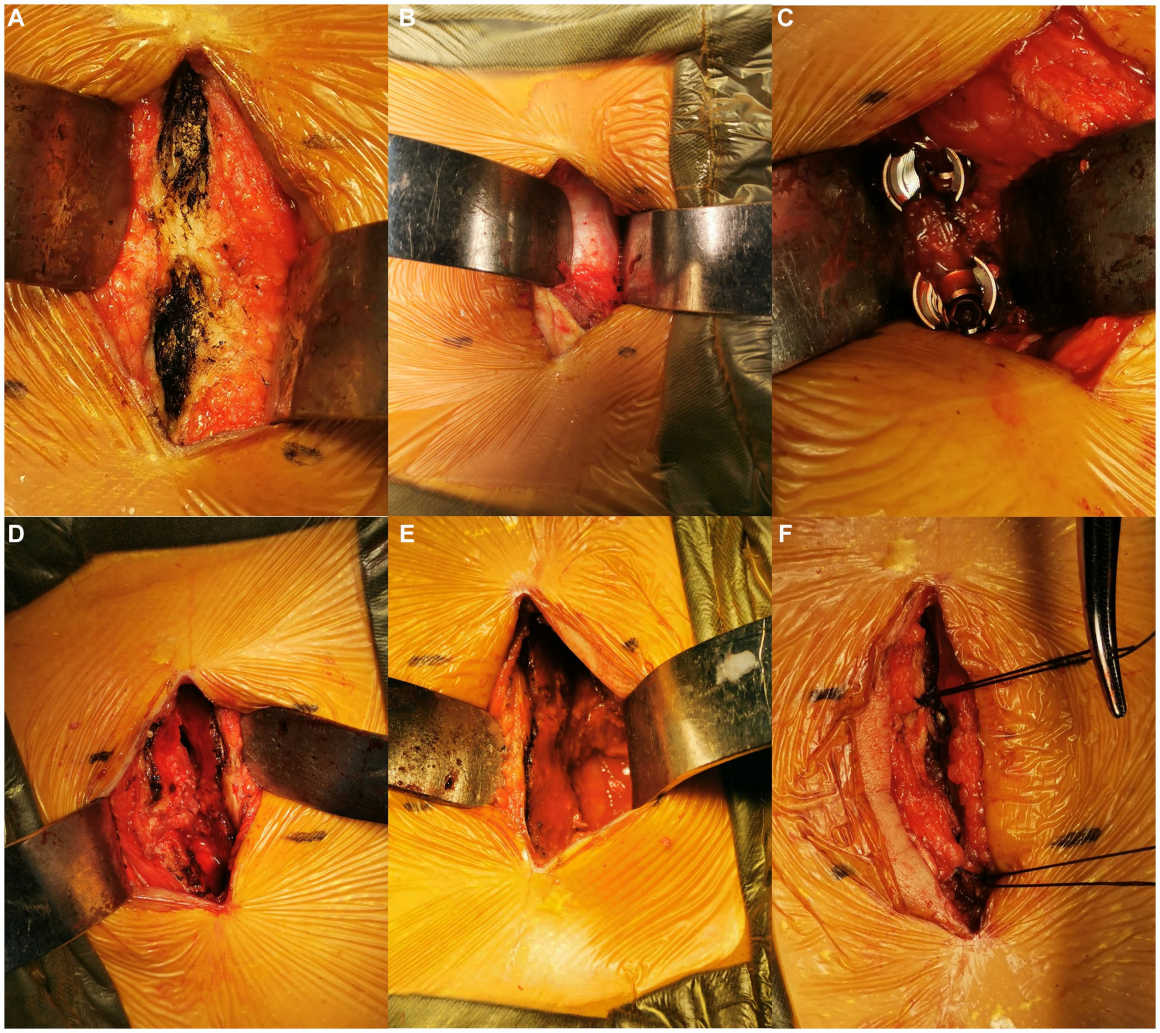
Intraoperative images. Exposure of the cranial and caudal spinous processes **(A)**. The Wiltse interval was identified **(B)** and bluntly separated for pedicle screw placement on the asymptomatic side **(C)**. The spinous process was longitudinally split and broken at the base on the symptomatic side, then laterally retracted **(D)**. The vertebral canal was exposed **(E)**. After the TLIF procedure, the spinous process was reattached by transosseous sutures **(F)**.

On the symptomatic side, the lateral half of the spinous process and interspinous ligaments were longitudinally split and broken at the base. Next, they were retracted laterally along with the attached paravertebral muscles. Unilateral laminectomy, facetectomy, and discectomy were then performed under direct vision, followed by a standard TLIF (2). Adequately high cage (GN Tech Co., Ltd., Chengdu, Sichuan, China) was used for interbody fusion, which could allow contralateral indirect decompression. After the insertion of pedicle screws through the surgical field on the symptomatic side, the screw and rod system were locked with mild pressure. The retracted spinous process half was reattached to the portion of the spinous process left in place using transosseous sutures. The interspinous ligaments were repaired using sutures (Figures 1, 2).

### Evaluation of clinical outcome

The Japanese Orthopedic Association (JOA) score for lumbar spinal disorders was determined before surgery and at the last follow-up (10). The recovery rate was calculated as (postoperative score − preoperative score)/(total score − preoperative score) × 100. Outcomes were defined according to recovery rate: excellent, recovery rate ≥ 75%; good, 50–74.9%; fair, 25–49.9%; and poor, <25%. Visual analog scale (VAS) scores for leg and back pain were also determined.

### Radiographic evaluation

Preoperative and final follow-up lateral radiographs were reviewed for evaluating global lumbar lordosis and segmental lordosis (11). Postoperative computed tomography was used to assess interbody fusion and bony union of the split spinous process. Approach-related paraspinal muscle damage was evaluated by measuring a cross-sectional area of the paraspinal muscles at the surgical level on the symptomatic (decompression) side on axial T2-weighted magnetic resonance imaging performed before surgery and at the last follow-up (Magnetom Avanto; Siemens, Munich, Germany). The muscle atrophy rate was calculated as (1 − total postoperative area/total preoperative area) × 100^8^. The paraspinal muscles on the asymptomatic side were used as a control (9). Radiographic evaluation was performed by two radiologists blinded to the study data. Any disagreements were resolved via discussion and consensus.

### Statistical analysis

Statistical analyses were performed using SPSS software version 18.0 (IBM Corp., Armonk, NY, USA). Pre- and postoperative clinical results were compared using the two-sample *t*-test. Muscle atrophy rate was reached between the symptomatic (decompression) side and the asymptomatic side using the paired *t*-test. All tests were two-sided. *P* < 0.05 was considered significant.

## Results

**Figure 3:**
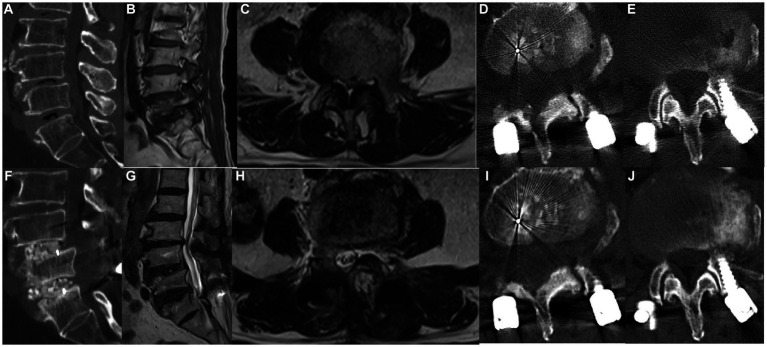
A 58-year-old woman underwent MHSPS TLIF for L4/5 lumbar spinal stenosis with instability and unilateral lower extremity pain. Preoperative sagittal computed tomography confirmed segmental instability **(A)**. Preoperative sagittal **(B)** and axial **(C)** T2-weighted magnetic resonance imaging shows lumbar spinal stenosis and instability. Three days after surgery, axial computed tomography shows the split spinous process of two levels **(D,E)**. Postoperative sagittal computed tomography 1 year after surgery shows a robust interbody fusion **(F)**. Axial **(G)** and sagittal **(H)** T2-weighted imaging 1 year after surgery shows good decompression and no marked difference in the cross-sectional area of the paraspinal muscles. Axial computed tomography 1 year after surgery shows a bony union of the spinous process of two levels **(I,J)**.

**Figure 4:**
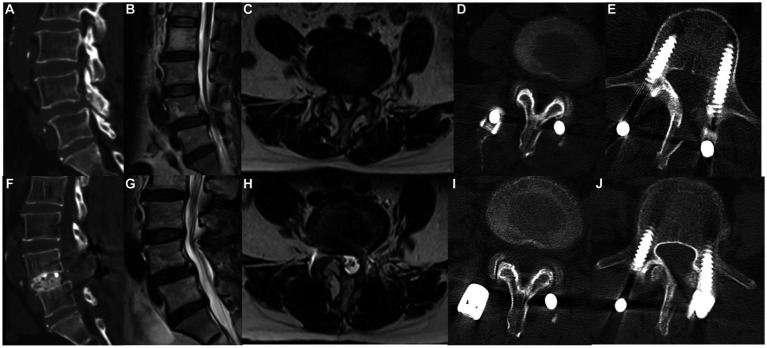
A 69-year-old man underwent MHSPS TLIF for L4/5 lumbar spinal stenosis with instability and unilateral lower extremity pain. Preoperative sagittal computed tomography demonstrates segmental instability **(A)**. Preoperative sagittal **(B)** and axial **(C)** T2-weighted magnetic resonance imaging shows L4/5 and L5/S1 spinal stenosis. Axial computed tomography 5 days after surgery shows the split spinous process at two levels **(D,E)**. Postoperative sagittal computed tomography demonstrates good interbody fusion **(F)**. Sagittal **(G)** and axial **(H)** T2-weighted imaging 1 year after surgery shows sufficient decompression and no marked difference in the cross-sectional area of the paraspinal muscles. Axial computed tomography 1 year after surgery demonstrates a bony union of the spinous process at two different levels **(I,J)**.

Sixty-five patients (43 men and 22 women) were included for analysis (Figures 3, 4). The mean age at the time of surgery was 52.27 ± 8.76 years. Preoperative diagnosis was degenerative disk disease with herniated nucleus pulposus in 39 patients, spondylolisthesis in 21, and lumbar stenosis in 5. The number of surgical levels was one in 49 patients and two in 15; three-level surgery was performed in one patient (Table 1).

**Table 1:** Patient demographic data.

	Patient
Mean age (years) mean ± SD	52.27 ± 8.76
Gender (M/F)	43/22
BMI (kg/m2) mean ± SD	22.9 ± 3.5
Disease course (months) mean ± SD	10.3 ± 5.5
Preoperative diagnosis	
Herniated nucleus pulposus	39
Spondylolisthesis	21
Lumbar stenosis	5
Level of fusion	
L3–L4	3
L4–L5	28
L5–S1	26

BMI, Body mass index.

The mean follow-up was 15.6 ± 3.7 months (range, 12–26). Mean operation time was 70.5 ± 15.6 min for one-level surgery, 120 ± 10.2 min for two-level surgery, and 160 min for three-level surgery.

**Figure 5:**
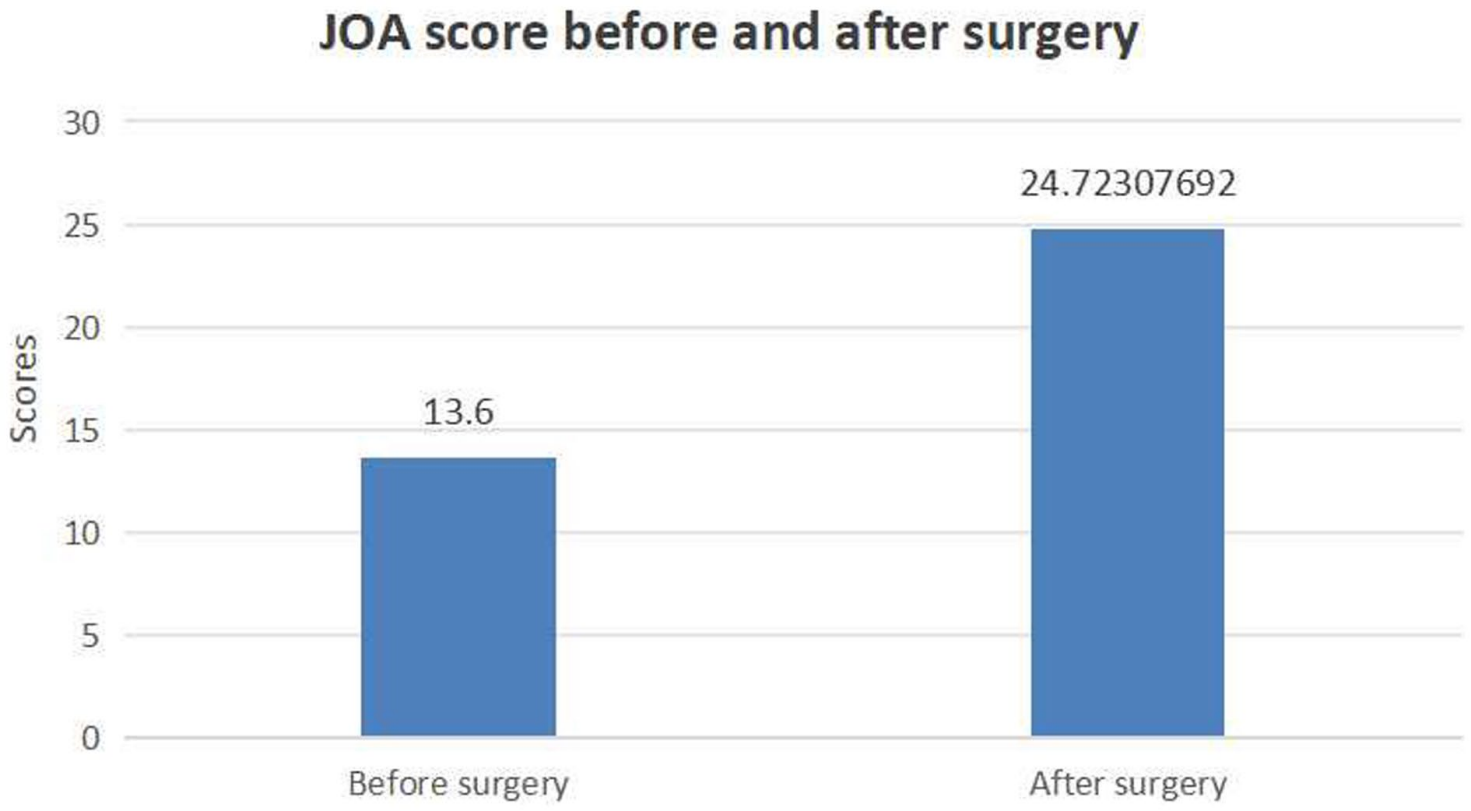
Bar graph showing the JOA score before and after surgery.

All 65 patients experienced symptom relief after surgery. JOA score was significantly higher at the last follow-up than before surgery (24.72 ± 3.34 vs. 13.6 ± 3.21; *p* < 0.001, Figure 5). The mean recovery rate was 68.2% ± 5.68%. The outcome was excellent, good, and fair in 22, 35, and 8 patients. The VAS score for low back pain was significantly lower at the last follow-up than before surgery (1.18 ± 0.99 vs. 3.09 ± 1.35; *p* < 0.001). The VAS score for leg pain was also significantly lower at the last follow-up than before surgery (1.13 ± 0.91 vs. 6.61 ± 1.23; *p* < 0.001).

**Figure 6:**
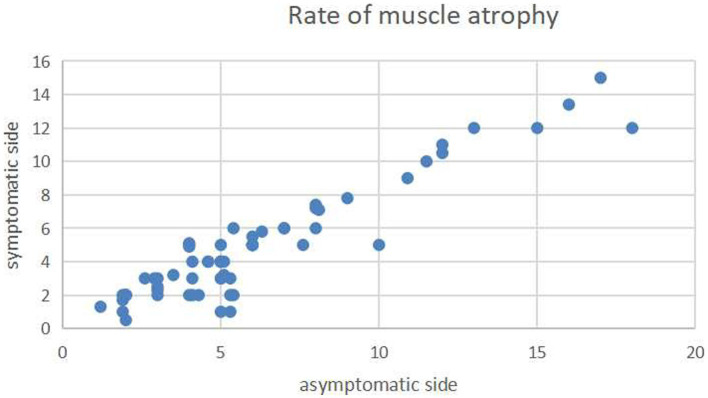
Scatterplot showing the mean atrophy rates of the paraspinal muscles on the axial T2-weighted imaging on the decompression (longitudinal axis) and nondecompression sides (horizontal axis). The atrophy rates did not significantly differ between the two sides.

Lumbar lordosis and segmental lordosis slightly increased from preoperative (42.49 ° ± 8.1° and 14.96° ± 5.12°) to preoperative (43.78 ° ± 7.29° and 16.2° ± 4.78°), but there was no significant difference (*t* = −0.955 and − 1.415, *p* = 0.341 and 0.159 respectively). Interbody fusion and fusion of the split spinous process were achieved in all patients. The mean paraspinal muscle atrophy rate did not significantly differ between the symptomatic (decompression) side (6% ± 3.8%) and the asymptomatic side (4.8% ± 3.3%) at the last follow-up (*p* = 0.071; Figure 6). Only one complication, a misplaced pedicle screw that required surgical repositioning, was observed; the patient experienced no neurological sequelae.

## Discussion

Unilateral TLIF with bilateral pedicle screw fixation is a standard surgical treatment for lumbar degenerative disease (2, 3). The conventional open approach is widely used because of its safety, straightforward technique, and good visualization (1). However, it requires extensive muscle stripping and retraction, which can cause iatrogenic muscle injury resulting in postoperative low back pain and/or failed back surgery syndrome (4, 5). MIS TLIF procedures are associated with less paraspinal muscle damage (6, 7). The Wiltse approach accesses the spine through the anatomic cleavage plane between the multifidus and longissimus muscles. Compared to the conventional approach, it causes less paraspinal muscle damage, blood loss, and back pain and is associated with better recovery of lumbar function (6, 12, 13). Although the Wiltse approach provides good exposure of the articular and transverse processes and enables pedicle screw placement, laminectomy can be challenging to perform because the medial multifidus impedes exposure of the lamina, especially in muscular or obese patients (6). A novel surgical retractor for specific use with the Wiltse TILF approach has recently been designed (6).

Watanabe et al. (8) compared lumbar stenosis patients who underwent lumbar laminectomy via the spinous process-splitting approach with those who underwent conventional laminectomy and reported that the latter approach provided adequate surgical working space and good visualization and caused less muscular injury than the conventional approach (8). Many other reports have confirmed the superiority of the spinous process-splitting approach (8, 9, 14–17). However, only some studies of this approach for TLIF have been previously reported. Mori et al. (18) studied patients who underwent single-level open pedicle screw fusion for degenerative spondylolisthesis (27 patients underwent the spinous process-splitting approach, and 26 underwent the conventional approach) and found the spinous process-splitting approach was less damaging to the paraspinal muscles; moreover, the patients who underwent the spinous process-splitting procedure had less low back discomfort 1 year after surgery. Our group recently compared a spinous process-splitting TLIF technique with conventional TLIF in patients with lumbar degenerative or isthmic spondylolisthesis and found that the spinous process-splitting technique allows for better visualization and a more expansive working space and minimizes damage to the paraspinal muscles (19). The present study modified the spinous process-splitting approach for unilateral TLIF to treat lumbar degenerative disease.

The MHSPS approach for TLIF combines the spinous process-splitting and Wiltse techniques. Unilateral laminotomy and pedicle screw insertion on the symptomatic (decompression) side are performed using the spinous process-splitting approach, while pedicle screw insertion on the asymptomatic side is performed via the Wiltse approach. Fusion was achieved in all patients and the lumbar lordosis was restored in our study. In addition, operation time was short and paraspinal muscle atrophy was negligible. Moreover, after surgery, the mean JOA score significantly increased and VAS scores for low back and leg pain significantly decreased.

Liu et al. (6) also reported improvements in JOA and VAS pain scores 12 months after Wiltse TLIF. In a study of 49 patients who underwent MIS TLIF for degenerative disk disease with a herniated disk, Schwender et al. (20) reported a decrease in VAS pain score from 7.2 before surgery to 2.1 at the last follow-up. The outcomes of our patients are comparable or superior to those reported in previous MIS TLIF studies.

MHSPS TLIF provides a clear surgical field and visualization, enabling a rapid operation. The mean operation time for one-level surgery in our study was 70.5 min; corresponding values were 120 min for two-level operations. These times are lower than those reported in previous MIS TLIF series (6, 12, 13). With the Wiltse and other MIS TLIF techniques, the multifidus is dissected and retracted medially before performing laminectomy. In contrast, with the MHSPS approach, the spinous process is split in the midline, and the lateral half is retracted laterally to expose the surgical field for laminectomy and discectomy. This approach can achieve similar functional outcomes with shorter operation time than the Wiltse TLIF and MIS TLIF without sacrificing visualization or working space.

Pedicle screw placement through the Wiltse approach is widely used and associated with less paraspinal muscle damage than the conventional approach (21, 22). Pedicle screws on the asymptomatic side in our study were placed through the Wiltse approach. Postoperative paraspinal muscle atrophy did not significantly differ between the asymptomatic and symptomatic sides where the decompression was performed. This suggests that any postoperative paraspinal muscle changes were minor. Mori et al. (18) used the spinous process-splitting approach for interbody fusion combined with bilateral pedicle screw insertion using the Wiltse approach in single-level operations and found the degree of paraspinal muscle injury was less than that seen with the conventional open approach. Our surgical technique was similar; however, we inserted pedicle screws on the symptomatic (decompression) side through the surgical incision, not via the Wiltse approach. A potential disadvantage of the spinous process-bilateral-splitting approach is that the force of the multifidus muscle cannot be transmitted to the spine because of the floating spinous process (8). Only the lateral half of the spinous process is broken and retracted with our MHSPS TLIF technique, which might preserve muscle function.

The MHSPS TLIF has several limitations. First, spinous process anatomy can vary between individuals. The reported mean width of the spinous processes at L4 and L5 is 9 mm (range, 3–18) (23) and splitting may be difficult in some. Second, the unilateral approach technique is unsuitable for patients with bilateral lower extremity symptoms; these patients should undergo a bilateral lumbar spinous process-splitting laminectomy approach. Third, the pedicle screw entry point on the symptomatic (decompression) side should be located more medially than on the opposite side because the split spinous process may limit the proper axial screw angle. Forth, although the spinous process and interspinous ligaments are longitudinally split and then repaired, the long-term functional outcome of damaging the posterior midline complex is uncertain (24). Finally, other limitations of this study includ the study type-case series without a control group, as well as the fact that it is debatable whether midline incision surgery can be considered minimally invasive. Future long-term, large-scale randomized controlled studies are warranted to investigate.

## Conclusion

MHSPS TLIF is an effective MIS surgical treatment for selected types of degenerative lumbar disease. This technique can achieve effective spinal decompression and interbody fusion. Its advantages include direct and adequate visualization, vast surgical working space, short operation time, and minimal muscle injury.

## Author contributions

GL: Writing – original draft. XZ: Writing – original draft. YD: Writing – review & editing. AA: Writing – review & editing. LH: Writing – review & editing. LM: Writing – review & editing. JQ: Writing – review & editing. JY: Writing – review & editing. SA: Writing – review & editing. OA: Writing – review & editing. YM: Writing – review & editing. HL: Writing – review & editing.

## References

[1] Harms J Rolinger H. A one-stager procedure in operative treatment of spondylolistheses: dorsal traction-reposition and anterior fusion. Z Orthop Ihre Grenzgeb. (1982) 120:343–7. doi: 10.1055/s-2008-1051624 7113376

[2] Foley KT Holly LT Schwender JD. Minimally invasive lumbar fusion. Spine. (2003) 28:S26–35. doi: 10.1097/01.BRS.0000076895.52418.5E 12897471

[3] Badlani N Yu E Kreitz T Khan S Kurd MF. Minimally invasive transforaminal lumbar interbody fusion (TLIF). Clin Spine Surg. (2020) 33:62–4. doi: 10.1097/BSD.0000000000000902 31625956

[4] Taylor H McGregor AH Medhi-Zadeh S Richards S Kahn N Zadeh JA. The impact of self-retaining retractors on the paraspinal muscles during posterior spinal surgery. Spine. (1976) 27:2758–62. doi: 10.1097/00007632-200212150-00004 12486343

[5] Onesti ST. Failed back syndrome. Neurologist. (2004) 10:259–64. doi: 10.1097/01.nrl.0000138733.09406.39 15335443

[6] Liu H Li J Sun Y Wang X Wang WJ Guo L. A comparative study of a new retractor-assisted WILTSE TLIF, MIS-TLIF, and traditional PLIF for treatment of single-level lumbar degenerative diseases. Orthop Surg. (2022) 14:1317–30. doi: 10.1111/os.13289, PMID: 35603557 PMC9251281

[7] Kim CH Easley K Lee JS Hong JY Virk M Hsieh PC. Comparison of minimally invasive versus open transforaminal interbody lumbar fusion. Global Spine J. (2020) 10:143S–50S. doi: 10.1177/2192568219882344, PMID: 32528799 PMC7263326

[8] Watanabe K Hosoya T Shiraishi T. Spinous process splitting laminectomy preserving posterior musculopligamentous complex for lumbar spinal canal stenosis. J Neurosurg Spine. (2003) 38:1401–8. doi: 10.3171/spi.2005.3.5.0405

[9] Chatani K. A novel surgical approach to the lumbar spine involving hemilateral split-off of the spinous process to preserve the multifidus muscle: technical note. J Neurosurg Spine. (2016) 24:694–9. doi: 10.3171/2015.5.SPINE141074, PMID: 26544596

[10] Fukui M Chiba K Kawakami M Kikuchi S Konno S Miyamoto M. Japanese orthopaedic association back pain evaluation questionnaire. Part 3. Validity study and establishment of the measurement scale: subcommittee on low back pain and cervical myelopathy evaluation of the clinical outcome committee of the Japanese orthopaedic association, Japan. J Orthop Sci. (2008) 13:173–9. doi: 10.1007/s00776-008-1213-y, PMID: 18528648 PMC2778667

[11] Ledesma JA Ottaway JC Lambrechts MJ Dees A Thomas TL Kurd MF. Early experience with uniplanar versus biplanar expandable interbody fusion devices in single-level minimally invasive transforaminal lumbar interbody fusion. Neurospine. (2023) 20:487–97. doi: 10.14245/ns.2244870.435 37401067 PMC10323343

[12] Jin YM Chen Q Chen CY Lyu J Shi B Yang C. Clinical research and technique note of TLIF by Wiltse approach for the treatment of degenerative lumbar. Orthop Surg. (2021) 13:1628–38. doi: 10.1111/os.13055, PMID: 34152699 PMC8313142

[13] Street JT Andrew Glennie R Dea N DiPaola C Wang Z Boyd M. A comparison of the Wiltse versus midline approaches in degenerative conditions of the lumbar spine. J Neurosurg Spine. (2016) 25:332–8. doi: 10.3171/2016.2.SPINE151018, PMID: 27104286

[14] Nomura H Yanagisawa Y Arima J Oga M. Clinical outcome of microscopic lumbar spinous process-splitting laminectomy: clinical article. J Neurosurg Spine. (2014) 21:187–94. doi: 10.3171/2014.4.SPINE1373 24878270

[15] Watanabe K Matsumoto M Ikegami T Nishiwaki Y Tsuji T Ishii K. Reduced postoperative wound pain after lumbar spinous process-splitting laminectomy for lumbar canal stenosis: a randomized controlled study. J Neurosurg Spine. (2011) 14:51–8. doi: 10.3171/2010.9.SPINE09933, PMID: 21142464

[16] Kanbara S Yukawa Y Ito K Machino M Kato F. Surgical outcomes of modified lumbar spinous process-splitting laminectomy for lumbar spinal stenosis. J Neurosurg Spine. (2015) 22:353–7. doi: 10.3171/2014.9.SPINE1457 25594729

[17] Kawakami M Nakao S Fukui D Kadosaka Y Matsuoka T Yamada H. Modified marmot operation versus spinous process transverse cutting laminectomy for lumbar spinal stenosis. Spine. (1976) 38:E1461–8. doi: 10.1097/BRS.0b013e31829ff4ae 23778375

[18] Mori E Okada S Ueta T Itaru Y Maeda T Kawano O. Spinous process-splitting open pedicle screw fusion provides favorable results in patients with low back discomfort and pain compared to conventional open pedicle screw fixation over 1 year after surgery. Eur Spine J. (2012) 21:745–53. doi: 10.1007/s00586-011-2146-2, PMID: 22237851 PMC3326135

[19] Liu G Hu L Shen F Hu Y Ma W. Clinical outcomes of transforaminal lumbar interbody fusion using a modified posterior spinous process-splitting approach for lumbar degenerative or isthmic spondylolisthesis: a prospective cohort study. J Neurosurg Spine. (2023) 19:1–7. doi: 10.3171/2023.4.SPINE2322 37209076

[20] Schwender JD Holly LT Rouben DP. Minimally invasive transforaminal lumbar interbody fusion (TLIF): technical feasibility and initial results. J Spinal Disord Tech. (2005) 18:S1–6. doi: 10.1097/01.bsd.0000132291.50455.d0 15699793

[21] Lu YJ Miao YM Zhu TF Wu Q Shen X Lu DD. Comparison of the Wiltse approach and percutaneous pedicle screw fixation under O-arm navigation for the treatment of thoracolumbar fractures. Orthop Surg. (2021) 13:1618–27. doi: 10.1111/os.13053, PMID: 34142446 PMC8313162

[22] Junhui L Zhengbao P Wenbin X Lu H Shengyun L Shunwu F. Comparison of pedicle fixation by the Wiltse approach and the conventional posterior open approach for thoracolumbar fractures, using MRI, histological and electrophysiological analyses of the multifidus muscle. Eur Spine J. (2017) 26:1506–14. doi: 10.1007/s00586-017-5010-1, PMID: 28247080

[23] Aylott CE Puna R Robertson PA Walker C. Spinous process morphology: the effect of ageing through adulthood on spinous process size and relationship to sagittal alignment. Eur Spine J. (2012) 21:1007–12. doi: 10.1007/s00586-011-2029-6, PMID: 21959943 PMC3337914

[24] Lee DY Lee SH. Spinous process splitting laminectomy for lumbar canal stenosis: a critical appraisal. Minim Invasive Neurosurg. (2008) 51:204–7. doi: 10.1055/s-2008-1073137, PMID: 18683110

